# Spotlights on ubiquitin-specific proteases in lung cancer: from multifaceted pathophysiological mechanisms to potential therapeutic targets

**DOI:** 10.7717/peerj.20702

**Published:** 2026-01-30

**Authors:** Xiaoyun Shen, Ruoqi Wang, Fei Su, Juanjuan Guo, Da Zhao, Fangyun Yuan, Tao Zhang, Xiaoming Hou

**Affiliations:** 1 Gansu University of Chinese Medicine, First School of Clinical Medicine, Gansu University of Chinese Medicine, Lanzhou, Gansu, China; 2 Department of Oncology, The First Hospital of Lanzhou University, The First Hospital of Lanzhou University, Lanzhou, Gansu, China

**Keywords:** Ubiquitin-specific proteases, Lung cancer, And deubiquitination

## Abstract

Lung cancer ranks as the leading cause of cancer-related mortality worldwide, characterised by complex molecular mechanisms and high therapeutic resistance. Ubiquitin-specific proteases, as core members of the deubiquitinating enzyme family, extensively participate in the initiation, progression, metastasis, and treatment resistance of lung cancer by regulating the stability of key proteins. Recent studies indicate that multiple Ubiquitin-Specific Proteases (USP) family members play pivotal roles in lung cancer: Ubiquitin-Specific Peptidase 7 (USP7) promotes proliferation and osimertinib resistance in non-small cell lung cancer by stabilising proteins such as ERβ, c-Abl, and KRAS; Ubiquitin-Specific Peptidase 9, X-linked (USP9X) mediates radiotherapy resistance by regulating KDM4C and REV1; USP10 influences cellular metabolism and chemotherapy sensitivity *via* PTEN/AKT/mTOR and HDAC6 pathways; Ubiquitin-Specific Peptidase 14 (USP14) enhances tumour migration by regulating β-catenin and Acf7 stability; Ubiquitin-Specific Peptidase 22 (USP22) amplifies tumour stem cell properties and suppresses ferroptosis *via* EGFR and BMI1 signalling; Ubiquitin-Specific Peptidase 35 (USP35) and Ubiquitin-Specific Peptidase 38 (USP38) respectively modulate apoptosis resistance and proliferation through BIRC3 and KLF5; while Ubiquitin-Specific Peptidase 39 (USP39) influences mitochondrial metabolism *via* PDHA, thereby promoting tumour growth. This paper systematically reviews the mechanisms of action of the aforementioned USPs in multiple key signalling pathways, including KRAS, TGF-β/SMAD, ferroptosis, and DNA damage repair. It further explores the potential value of small-molecule inhibitors targeting USPs (such as P5091, IU1, and gentiopicroside) in reversing drug resistance, inducing apoptosis, and enhancing immunotherapy. Nevertheless, current research remains subject to certain limitations, including insufficient systematic and synergistic understanding of USP family members’ functions, poor inhibitor selectivity and preclinical toxicity concerns, as well as unresolved functional heterogeneity across different molecular subtypes of lung cancer. This paper reviews the molecular mechanisms and targeting strategies of USPs in lung cancer based on a systematic literature search of PubMed and Web of Science databases. It further explores their potential applications in precision lung cancer therapy, providing theoretical foundations and directional guidance for future research.

## Introduction

According to data from the International Agency for Research on Cancer (IARC), China recorded approximately 816,000 new lung cancer cases in 2020, with around 715,000 deaths attributed to the disease, this represents 23.8% of all cancer-related fatalities (Thai et al., 2021). Despite advances in targeted and immunotherapy improving survival for some patients, the National Cancer Institute’s Surveillance, Epidemiology, and End Results (SEER) database indicates that an estimated 127,070 individuals died from lung cancer in 2023, accounting for 20.8% of all cancer-related deaths, it ranks third in incidence among cancer diagnoses, accounting for 12.2% of all cancer cases, trailing only breast and prostate cancer, prognosis remains poor following diagnosis, with a five-year relative survival rate of merely 25.4% (Roelofsz & Rampon, 2024). High metastasis rates, tumour heterogeneity, and therapeutic resistance remain significant clinical challenges. However, with the progressive shift towards precision medicine, the application of targeted therapies, immunotherapies, and cell therapies has improved survival outcomes for lung cancer patients. During the initiation and progression of lung cancer, Ubiquitin-Specific Proteases (USPs) may influence key cellular processes—including proliferation, metastasis, and drug resistance—by regulating the ubiquitination status of numerous critical proteins. Consequently, in-depth investigation of USPs holds promise for identifying novel therapeutic strategies and targets for lung cancer treatment.

**Figure 1:**
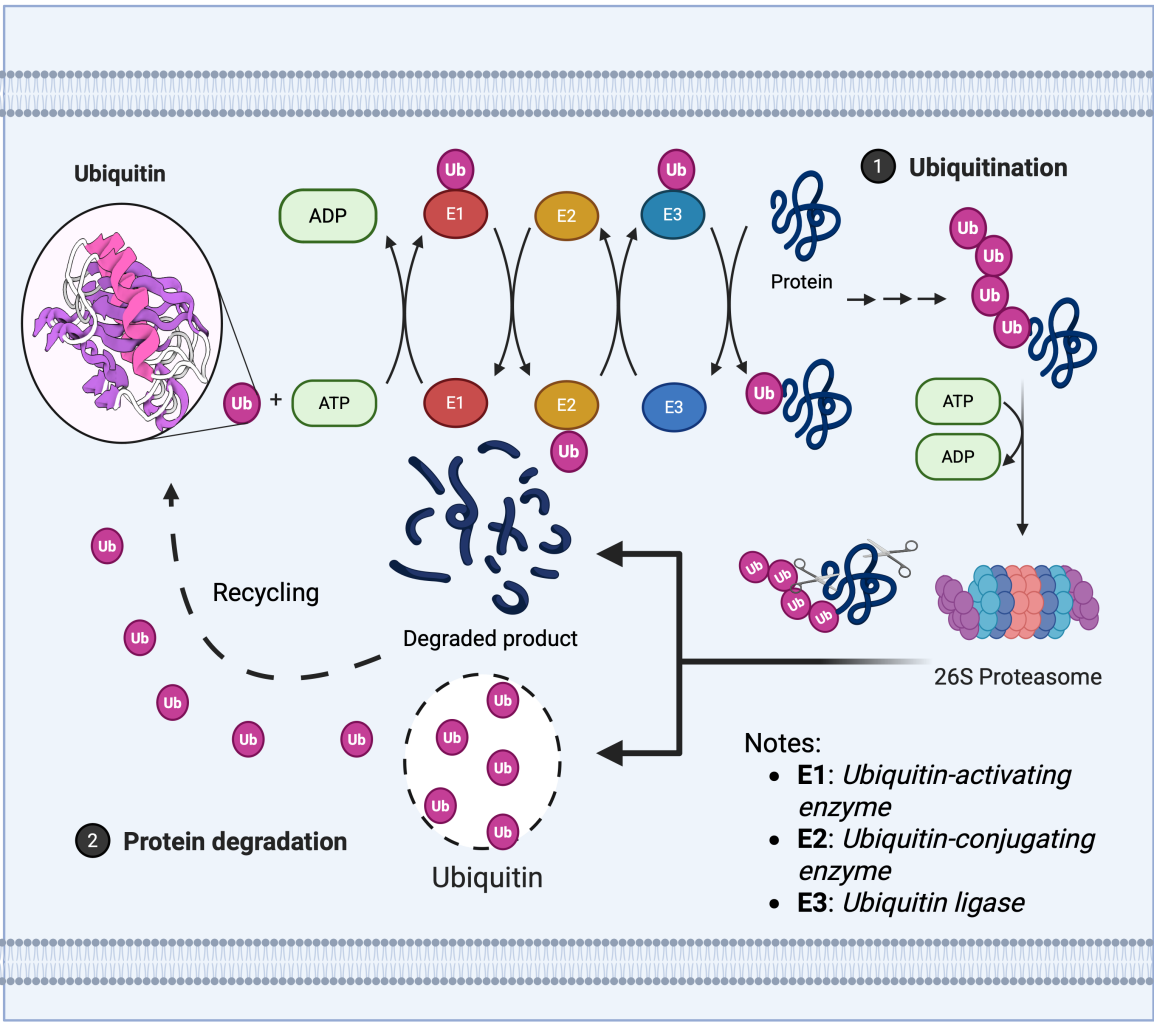
Ubiquitin enzyme cascade regulates protein degradation. The ubiquitin-mediated protein degradation process primarily comprises three sequential steps: 1. Ubiquitination modification: (A) The E1 ubiquitin-activating enzyme hydrolyses ATP to activate ubiquitin, transferring it to the E2 ubiquitin-conjugating enzyme. (B) The E3 ubiquitin ligase recognises the target protein and catalyses the covalent linkage of a polyubiquitin chain to the substrate, thereby marking it for degradation. 2. Proteasome recognition: Proteins labelled with ubiquitin are recognised and captured by the 26S proteasome. 3. Degradation and recycling: (A) The proteasome unfolds and degrades target proteins in an ATP-dependent manner, breaking them down into short peptides or amino acids. (B) Ubiquitin molecules are released and recycled, whilst degradation products can be reused by the cell.

The maintenance of protein homeostasis relies upon the dynamic regulation of the UPS, whose core mechanism encompasses two meticulously interlinked post-translational modification processes: The E1 (ubiquitin activase), E2 (ubiquitin conjugase), and E3 (ubiquitin ligase) cascade covalently attaches ubiquitin molecules to target proteins, mediating their degradation or functional regulation, as illustrated in Fig. 1 (Franić, Zubčić & Boban, 2021).

**Figure 2:**
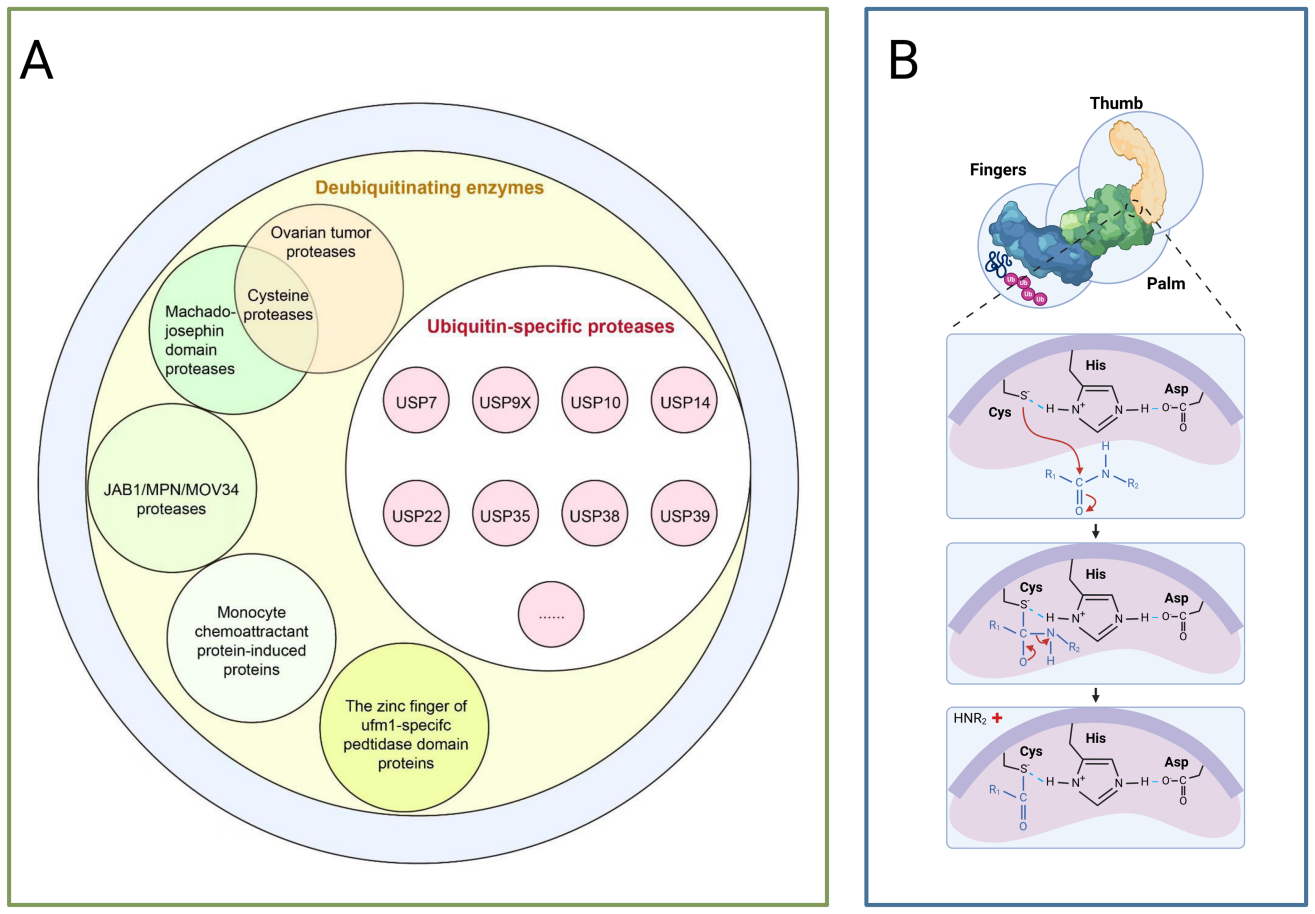
The deubiquitinating enzyme system and its members. (A) Protease classification: (1) Deubiquitinating enzymes: including ovarian tumour protease, cyclin protease, and mechanoprotease. (2) Ubiquitin-specific proteases (USP family): members include Ubiquitin-Specific Peptidase 7 (USP7), Ubiquitin-Specific Peptidase 9, X-linked (USP9X), Ubiquitin-Specific Peptidase 10 (USP10), and Ubiquitin-Specific Peptidase 14 (USP14). (3) JAB1/MPN/MOV34 protease family: includes monocyte chemotactic protein-induced protein (MCPIP) and zinc finger domain proteases. (B) Protease catalytic domain: Catalyses a nucleophilic attack by the catalytic cysteine on the dipeptidyl bond between the C-terminal glycine of ubiquitin and the substrate lysine, forming a thioester intermediate. Ubiquitin is subsequently released *via* hydrolysis. The sulphur atom of the thiol anion (−S^−^) of Cys points towards the carbon atom of the carbonyl group (C=O), with this arrow representing the nucleophilic attack. The carbon atom transitions from sp^2^ hybridisation (planar) to sp^3^ hybridisation (tetrahedral), causing the oxygen atom to acquire a negative charge. Subsequently, the peptide bond breaks, and finally, the amino group (−NH_2_) of the substrate lysine departs with an electron pair.

Deubiquitination, conversely, is mediated by deubiquitinating enzymes (DUBs) which reversibly remove ubiquitin tags, this process maintains system homeostasis by balancing ubiquitin signalling pathways, thereby protecting target proteins from degradation, recycling free ubiquitin molecules, and editing ubiquitin chain structures (such as K48/K63 linkage conversion) (Clague et al., 2013; Zheng et al., 2023). Based on catalytic domain characteristics, DUBs are primarily categorised into seven classes as depicted in Fig. 2A: USPs, ubiquitin carboxy-terminal hydrolases (UCHs), ovarian tumour proteases (OTUs), Machado-Joseph domain proteases (MJDs), monocyte chemotactic protein-induced proteins, novel motifs interacting with ubiquitin-containing DUB families, and zinc fingers of ubiquitin-like modification-specific protease domain proteins (UFM1s), each class regulates critical physiological or pathological processes through unique mechanisms, such as DNA repair, inflammatory responses, and neurodegenerative diseases (Li & Reverter, 2021; Ray & Mukherjee, 2024).

USPs constitute the largest subfamily of DUBs, comprising over 50 members, all possess a highly conserved USP domain, whose catalytic structure exhibits a distinctive ‘right-handed’ configuration, this configuration is formed by the palm, thumb, and finger subdomains (Cruz, Soares & Correia, 2021). The catalytic centre resides between the palm and thumb, comprising a highly conserved catalytic triad of cysteine (Cys), histidine (His), and aspartic acid (Asp), during catalysis, the cysteine residue first attacks the heteropeptide bond formed between the C-terminal glycine of ubiquitin and the modified substrate, yielding a covalent thioester intermediate, subsequently, a water molecule completes a nucleophilic attack with the assistance of the histidine-aspartic acid pair, ultimately releasing the free ubiquitin molecule and restoring the substrate protein to its unmodified state,the finger domain is responsible for recognising distant ubiquitin molecules, conferring substrate specificity upon USPs (as shown in Fig. 2B) (Chen, Liu & Zhou, 2021). Moreover, USPs exert a dual role in carcinogenesis by regulating the stability of key proteins such as p53, NF-κB, and β-catenin through deubiquitination (Chen, Liu & Zhou, 2021).

The functions of USPs extend far beyond the field of cancer. As the most extensive family of deubiquitinating enzymes (DUBs), USPs play a pivotal role in the onset and progression of numerous human diseases by regulating the stability of key proteins, it also plays a significant role in neurodegenerative diseases, viral infections, autoimmune disorders, and metabolic diseases (Alsohime et al., 2020; Jean-Charles et al., 2018; Jolly et al., 2020; Okarmus et al., 2024; Zhang et al., 2020). Table 1 outlines the functions of selected USP family members in other major diseases. The central role of USPs in multiple disease pathways not only underscores their biological significance but also offers substantial prospects for translational research into their potential as therapeutic targets, the subsequent discussion will focus specifically on the mechanisms of action and therapeutic potential of USP family members in the context of lung cancer (Alsohime et al., 2020; Jean-Charles et al., 2018; Jolly et al., 2020; Okarmus et al., 2024; Zhang et al., 2020).

**Table 1:** Overview of the function of selected USP members in other major diseases. This table summarises the key functions of five representative ubiquitin-specific proteases (Ubiquitin-Specific Peptidase 20 (USP20), USP7, Ubiquitin-Specific Peptidase 18 (USP18), Ubiquitin-Specific Peptidase 30 (USP30), and USP9X) in major diseases including cardiovascular disorders, neurodegenerative diseases, viral infections/autoimmune diseases, Parkinson’s disease, and neurodevelopmental disorders. By deubiquitinating specific substrates, these enzymes participate in regulating core biological processes such as inflammatory responses, protein homeostasis, interferon signalling, mitochondrial autophagy, and neurodevelopment.

USP members	Related diseases	Primary functions	References
USP20	cardiovascular disease	By deubiquitinating signalling intermediates such as RIPK1, it suppresses inflammation in smooth muscle cells and alleviates atherosclerosis	Jean-Charles et al. (2018)
USP7	Neurodegenerative diseases	USP7 stabilises SMAD2 by deubiquitinating the E3 ubiquitin ligase NEDD4L, thereby activating autophagy, promoting the clearance of misfolded proteins, regulating proteostasis, and counteracting ALS-associated protein toxicity	Zhang et al. (2020)
USP18	Viral infection/autoimmune disease	USP18 is a key negative regulator of type I interferon signalling, inhibiting the pathway by blocking the interaction between JAK1 and IFNAR2. Its absence or dysfunction leads to fatal inflammation	Alsohime et al. (2020)
USP30	Parkinson’s disease	USP30 is a mitochondrial-associated deubiquitinating enzyme that negatively regulates mitochondrial autophagy; its inhibition accelerates the clearance of damaged mitochondria, balances oxidative stress, and improves the health of Parkin-deficient neurons	Okarmus et al. (2024)
USP9X	neurodevelopmental disorder	USP9X is a deubiquitinating enzyme on the X chromosome that evades X-chromosome inactivation, regulating protein homeostasis. Its loss of function causes neurodevelopmental disorders in both males and females, with distinct mutation types correlating to different phenotypes	Jolly et al. (2020)

## Survey Methodology

The literature search for this paper was systematically conducted in the PubMed and Web of Science databases. The search timeframe was set from the inception of the databases to June 2025, with a particular focus on research articles and review papers published in the last decade (since 2015) to reflect the latest advances in the field. The search strategy employed the following terms and Boolean operators: (‘ubiquitin-specific protease’ OR ‘USP’) AND (‘lung cancer’ OR ‘non-small cell lung cancer’ OR “NSCLC” OR ‘small cell lung cancer’ OR ‘SCLC’). This was further expanded to include relevant mechanisms and phenotypes such as: “drug resistance” “metastasis” “ferroptosis” “DNA damage repair” “proliferation”, apoptosis and kinase signalling. After excluding duplicates and articles with weaker relevance, a final selection of 93 papers was incorporated into this review, forming its literature foundation.

## The Impact of Diferent Usps on Lung Cancer

### USP7

Ubiquitin-Specific Peptidase 7 (USP7) comprises two domains: an N-terminal TRAF domain and a C-terminal ubiquitin-like domain, the TRAF domain of USP7 binds to the [P/A/E]-X-X-S motif in multiple substrates, such as p53, MDM2, MDM4, pyruvate kinase M2, Epstein-Barr nuclear antigen 1, microchromosome maintenance complex-associated protein, F-box protein 38, tripeptidyl peptidase 1, and ubiquitin-conjugating enzymes E2 and E1,it plays a crucial role in maintaining normal protein function and preventing disease, participating in the construction and regulation of protein interaction networks, and sustaining cellular genomic stability and survival (Carreira et al., 2023; Choi et al., 2020; Honda, Okumura & Chihara, 2023; Jagannathan et al., 2014; Park & Baek, 2023; Penev et al., 2022; Qi et al., 2020).

Research has revealed that USP7 stabilises the oestrogen receptor β (ERβ) through deubiquitination, inhibits the SUMOylation of peroxiredoxin-3 (PRDX3), and mitigates reactive oxygen species (ROS) accumulation, this mechanism promotes the development of osimertinib resistance in non-small cell lung cancer (NSCLC), offering novel insights for overcoming osimertinib resistance challenges (Meng et al., 2024). He et al. (2023) indicate that USP7, acting as a deubiquitinating enzyme for c-Abl, induces the accumulation of c-Abl in the cytoplasm, promotes its phosphorylation, and stabilises hexokinase 2 (HK2), this process drives glycolysis in non-small cell lung cancer tumour cells, ultimately facilitating tumour cell proliferation and metastasis. Huang et al. (2021) demonstrated that USP7 promotes the autoregulation of SMAD3 through its deubiquitinating enzyme activity, thereby inhibiting the proliferation of p53-deficient lung cancer cells. Furthermore, studies have indicated that USP7 stabilises KRAS through deubiquitination, thereby stimulating the proliferation of NSCLC cells, moreover, when USP7 inhibitors are combined with KRAS inhibitors, they exhibit a synergistic inhibitory effect on NSCLC cells resistant to KRAS inhibitors (Huang et al., 2024). Further research indicates that USP7, by binding to the PVDS motif of Raf-1, can deubiquitinate multiple polyubiquitin chains on Raf-1, thereby reducing the extent of its threonine phosphorylation, this process inhibits the activity of the ERK1/2 signalling pathway, consequently arresting the proliferation of lung adenocarcinoma cells (Park, Hwang & Baek, 2022).

### USP9X

Ubiquitin-Specific Peptidase 9, X-linked (USP9X) is an X-linked USP protein associated with embryonic and neural development (Kugler & Lasko, 2009). It possesses unique functional characteristics, capable of both promoting and inhibiting apoptosis, with these functions mediated by its deubiquitination effects on key components of the apoptotic signalling network (Park & Baek, 2022; Sulkshane et al., 2021). Previous studies have demonstrated that USP9X modulates the expression of stress-responsive and pro-apoptotic kinases, thereby initiating the JNK apoptotic signalling cascade (Niu et al., 2022; Park & Baek, 2022). In cancer cells, USP9X also exerts regulatory control over numerous tumour functions, encompassing cell adhesion, cell polarity, cell death, and inflammatory processes (Gao et al., 2024; Moon et al., 2021).

Research indicates that USP9X serves as a crucial interacting partner for histone demethylase 4C (KDM4C), capable of deubiquitinating KDM4C and maintaining its stability, this subsequently reduces the level of histone H3 lysine 9 trimethylation (H3K9me3) at the transforming growth factor-β2 (TGF-β2) promoter, ultimately promoting radiotherapy resistance in lung cancer cells by activating the SMAD/ATM/Chk2 signalling pathway (Jie et al., 2021). Moreover, REV1 plays a pivotal role in the development of radiotherapy resistance in lung cancer, its abnormally high expression is triggered by deubiquitination mediated by USP9X,specifically, acting as a scaffold protein, REV1 promotes the binding of Rad18 to CTH and accelerates CTH degradation,this induces abnormalities in the tumour metabolic microenvironment, ultimately leading to the emergence of radiotherapy resistance (Chen et al., 2024).

### USP10

Ubiquitin-Specific Peptidase 10 (USP10), as a cysteine protease, primarily mediates the thiol-dependent hydrolysis of ester, thioester, or peptide bonds formed by the carboxyl-terminal glycine residue of ubiquitin, through this hydrolysis process, the Ub moiety is removed from the target protein (Meyer et al., 2020). Among numerous targets, the tumour protein p53, cystic fibrosis transmembrane conductance regulator, adenosine monophosphate (AMP)-activated protein kinase alpha, silencer protein 6, and nuclear factor κB essential regulator constitute key targets of USP10, these targets are profoundly involved in vital cellular processes, which fully accounts for USP10’s pivotal role in cellular metabolism, signal transduction, and tumourigenesis (Tao et al., 2022; Wang et al., 2023c).

He et al. (2021b) demonstrated that USP10 activates phosphatase and tensin homolog (PTEN) by removing the K63-linked polyubiquitin chain from PTEN, thereby inhibiting the AKT/mTOR signalling pathway and ultimately suppressing the proliferation of NSCLC tumour cells. Additionally, studies have reported that USP10, acting as a deubiquitinating enzyme for histone deacetylase 6 (HDAC6), regulates the stability of HDAC6,when USP10 is knocked down or inhibited, cells harbouring mutant or loss-of-function p53 become sensitive to cisplatin (Zhang et al., 2021). Moreover, USP10 promotes the proliferation, migration, and invasion of NSCLC cells by deubiquitinating and thereby stabilising the eukaryotic initiation factor 4G1 (EIF4G1), leading to increased EIF4G1 levels (Li et al., 2024a).

### USP14

Ubiquitin-Specific Peptidase 14 (USP14), comprising 494 amino acids and two distinct domains, features a ubiquitin-like domain at its N-terminus that regulates proteasome activity, whilst its C-terminal USP domain governs the deubiquitinating enzyme activity of USP14 (Boughton et al., 2021). Research indicates that USP14 activity is subject to stringent regulation, with its regulatory mechanisms encompassing association with the proteasome and various post-translational modifications, such as phosphorylation (Wang et al., 2021a; Wang, Zhang & Wu, 2023b). Once USP14 becomes dysregulated, it may trigger a series of pathological abnormalities, such as cancer, neurodegenerative diseases, autophagy, immune responses, and viral infections (Wang et al., 2021a).

Research has revealed that following interaction between ceramide synthase 1 (CERS1) and USP14, the PI3K/AKT/mTOR signalling pathway is downregulated, thereby inhibiting the proliferation, migration, invasion, and brain metastasis capabilities of NSCLC cells (Yan et al., 2021). Additionally, research indicates that in lung adenocarcinoma, overexpression of USP14 promotes the accumulation of β-catenin (β-catenin 17), thereby driving tumour cell proliferation, furthermore, USP14 regulates lung tumourigenesis *via* apoptotic and autophagic pathways and is critically important for NSCLC migration, this process is achieved by deubiquitinating actin cross-linking family protein 7 (Acf7) and maintaining its stability (Lee et al., 2023). Moreover, in cisplatin-resistant lung cancer, USP14 exhibits high expression levels and correlates with poor prognosis. Reducing USP14 expression enhances cisplatin’s antitumour efficacy against lung cancer cells, whilst aptamers—siRNA nano-zinc carriers represent an innovative drug delivery system for targeted therapy, they effectively suppress resistance in cisplatin-resistant lung cancer cells and demonstrate antitumour efficacy *in vivo* (Zhao et al., 2023).

### USP22

Ubiquitin-Specific Peptidase 22 (USP22) functions as a subunit of the human Spt-Ada-Gcn5—acetyltransferase (SAGA) complex, plays a pivotal role in histone modification, it facilitates histone acetylation *via* GCN5 whilst simultaneously deubiquitinating histones H2B and H2A through its own activity, and is deeply involved in regulating gene transcription (Grasser, Rubio & Barneche, 2021; Zhou et al., 2021). In normal tissues, USP22 actively participates in the growth, development, and phenotypic conversion processes of T cells and B cells, in the context of cancer, USP22 may induce alterations in the immune microenvironment and has been identified as a significant biomarker for cancer mortality, being closely associated with cancer recurrence, metastasis, and patient survival outcomes (Guo et al., 2022).

Research indicates that USP22 expression is promoted by Activator Protein 2 (AP2) and c-Myc, with AP2 acting as a key transcription factor driving USP22 expression, this may partially occur through upregulating USP22’s transcriptional levels, thereby enhancing the malignancy of NSCLC (Sun et al., 2022). USP22 not only enhances EGFR signalling activity but also promotes resistance to EGFR-TKIs in lung cancer patients, furthermore, USP22 drives the acquisition of stem cell-like characteristics in NSCLC cells by regulating BMI1 signalling (Thai et al., 2021). Further research has demonstrated that, through the establishment of a mouse xenograft model, USP22 overexpression induces gefitinib resistance, moreover, *in vitro* experiments show that deubiquitination of the mouse homologue of the double microbody 2 gene (MDM2) inhibits ferroptosis in NSCLC cells (Lu, Li & Xu, 2024a).

### USP35

Ubiquitin Specific Peptidase 35 (USP35) belongs to the peptidase C19 family and plays a crucial role in the execution of multiple cellular functions, recent research evidence indicates that USP35 is deeply involved in regulating the cell cycle, determining cell fate, and driving cancer progression, specifically, USP35 effectively modulates the mitotic process by deubiquitinating Aurora B kinase, thereby preventing its degradation by the APC/CDH1 complex (Park et al., 2023). According to relevant reports, USP35 can also regulate the abundance of mitochondrial fusion protein 2 (MFN2) and PARK2-mediated mitochondrial autophagy (Qin et al., 2022). Moreover, USP35 inhibits NFκB activation by stabilising ABIN-2, thereby impeding tumour growth (Zou et al., 2024).

Research indicates that knocking down USP35 induces a series of effects: not only does it inhibit the growth, colony formation, and tumour invasion of lung cancer cells, but it also accelerates ferroptosis-mediated lipid peroxidation and intracellular iron overload, this leads to reduced glutathione (GSH) levels and decreased glutathione peroxidase 4 (GPX4) activity, while simultaneously enhancing the sensitivity of lung cancer cells to cisplatin and paclitaxel (Tang et al., 2021). Furthermore, the stability of USP35 is regulated or maintained by fusin (FUS), USP35 interacts with vascular endothelial growth factor A (VEGFA), and FUS-stabilised USP35 promotes the growth, invasion, and angiogenesis of NSCLC cells by deubiquitinating VEGFA (Li et al., 2024c). Liu et al. (2022) demonstrated that USP35 directly associates with the apoptosis-inhibiting protein BIRC3, modifying it *via* its deubiquitinating enzyme activity to prevent proteasomal degradation, by stabilising BIRC3, USP35 inhibits cisplatin-induced apoptosis in NSCLC cells, ultimately conferring resistance to cisplatin. Wang et al. (2022) indicate that in NSCLC, USP35 alleviates endoplasmic reticulum stress-induced apoptosis by stabilising ribosomal binding protein 1.

### USP38

Recombinant Ubiquitin-Specific Peptidase 38 (USP38) was initially identified as a negative regulator of type I interferon signalling, acting by modulating the ubiquitination of TANK-binding kinase 1 (He et al., 2021a). Simultaneously, it enhances its own stability through automated deubiquitination (Hu et al., 2024). Research indicates that USP38 possesses the capacity to recognise multiple types of ubiquitin modifications, encompassing K11 (Wang et al., 2024), K27 (Yi et al., 2022), K33 (Zhang et al., 2024), K48 (Zhang et al., 2024) and K63 (Zhan et al., 2020). Moreover, USP38 exerts crucial regulatory functions across multiple pathways, including inflammatory responses, antiviral defence, cell proliferation, migration, invasion, DNA damage repair, and chemotherapy resistance (Huang et al., 2022; Wang et al., 2023a; Wang et al., 2021b; Yi et al., 2022; Zheng et al., 2022).

Previous studies have demonstrated that USP38 promotes the proliferation of lung cancer cells by stabilising the c-myc protein (Xu et al., 2021). Zhang et al. (2024) noted that in LUAD cells, USP38 participates in maintaining the stability of the transcription factor KLF5 through its deubiquitinating activity, thereby promoting tumour cell proliferation, specifically, USP38 knockdown increases KLF5 ubiquitination levels, whereas USP38 overexpression reduces KLF5 ubiquitination, this occurs because USP38 removes K33- and K48-linked ubiquitin chains from KLF5, thereby inhibiting proteasomal degradation of the protein and ultimately increasing KLF5 expression, consequently, the USP38/KLF5 axis holds promise as a potential therapeutic target for lung adenocarcinoma.

### USP39

Ubiquitin-Specific Peptidase 39 (USP39) functions as a pivotal splicing factor, playing a crucial role in cellular activities, it is essential for maintaining the integrity of the mitotic spindle checkpoint and regulates the mRNA levels of Aurora B (Francois et al., 2022). Moreover, within NSCLC tissues, USP39 further stabilises the pyruvate dehydrogenase E1α subunit within the pyruvate dehydrogenase complex through deubiquitination, this action regulates the conversion of pyruvate into the tricarboxylic acid (TCA) cycle, ultimately influencing mitochondrial respiration, cellular proliferation, and tumour growth (Becirovic et al., 2024).

## Development of usp Inhibitors

**Figure 3:**
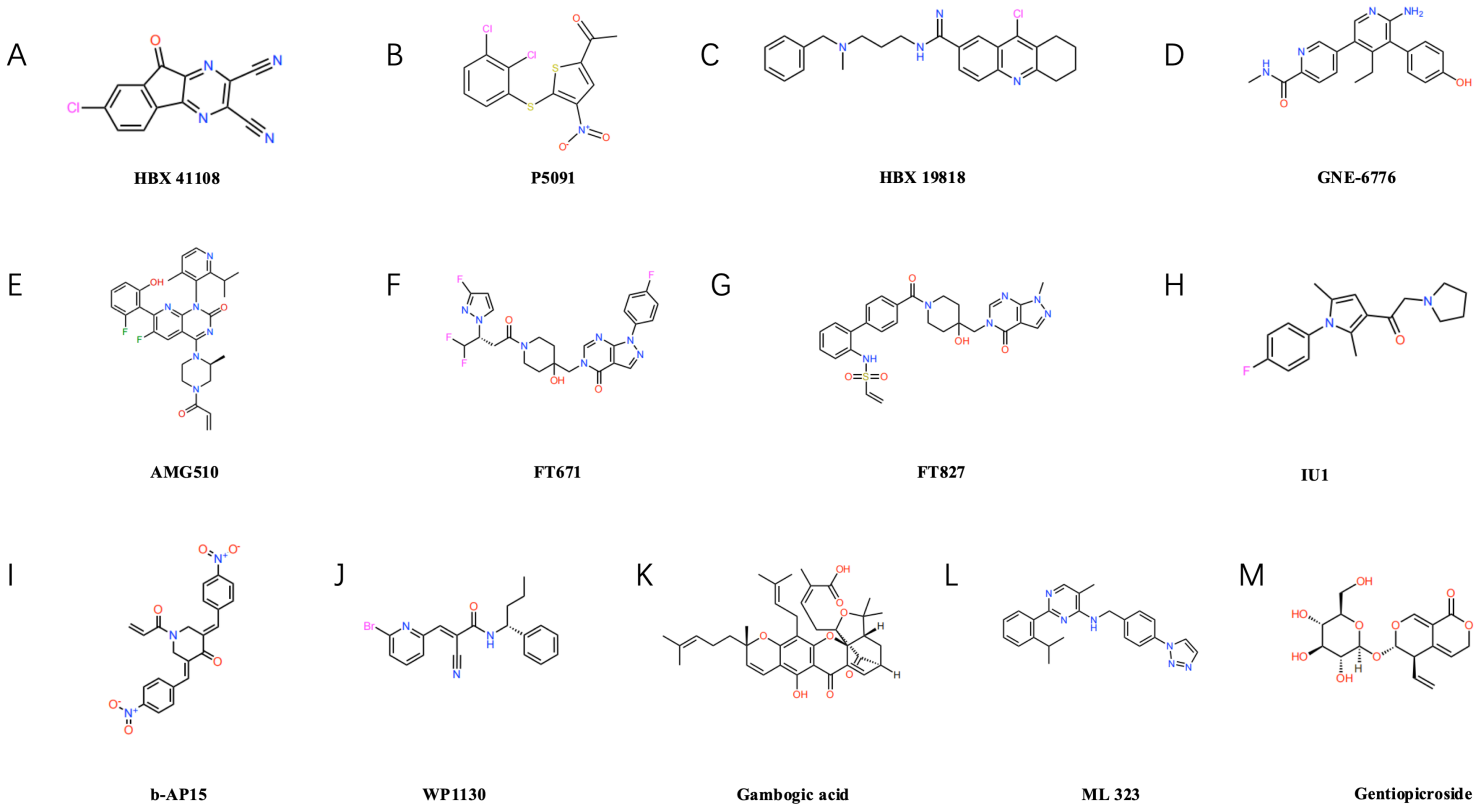
Structural formulae of selected small-molecule inhibitors targeting ubiquitin-specific proteases (USPs). USP7 inhibitors: such as P5091 (B), HBX19818 (C), GNE-6776 (D), FT671 (F), FT827 (G). USP14 inhibitors: such as IU1 (H). Inhibitors of other USP family members: Including b-AP15 (I) targeting USP14/UCHL5, USP9X inhibitors WP1130 (J) and gambogic acid (K), USP1-UAF1 complex inhibitor ML323 (L), and USP22-specific inhibitor gentiopicrin (M).

USPs, as the most numerous and functionally diverse family within the deubiquitinating enzyme group, saw the development of their inhibitors commence in the early 21st century. The first reported USP inhibitor was the small molecule compound HBX41108 (as shown in Fig. 3A), targeting USP7/HAUSP, discovered in 2009 by a Harvard University team, it was initially employed to investigate USP7’s regulatory role in the p53-MDM2 pathway, although not directly utilised in cancer therapy, it laid the groundwork for subsequent USP-targeting drug development (Colland et al., 2009). Subsequently, with the deepening understanding of the mechanism of action of USPs in tumours, multiple members of the USP family have emerged as key targets in the development of anti-tumour drugs.

### Mechanism of action and selectivity profile of USPs inhibitors

USPs inhibitors competitively or non-competitively block interactions with ubiquitin chains or ubiquitinated substrates by binding with high affinity to the catalytic active site of the target enzyme (Kategaya et al., 2017). This inhibitory effect prevents the efficient removal of ubiquitin tags from substrate proteins, leading to persistently elevated ubiquitin levels. Its downstream effects manifest primarily in two ways: firstly, for proteins primarily degraded *via* the proteasome pathway, sustained ubiquitination promotes their recognition and degradation by the 26S proteasome, thereby reducing the intracellular abundance of oncogenic proteins; Secondly, it disrupts numerous physiological processes dependent on reversible ubiquitination, such as DNA damage repair, cellular metabolism, and immune signalling pathways,this ultimately induces cell cycle arrest, apoptosis, or enhances the efficacy of chemotherapy/immunotherapy (Mani & Gelmann, 2005). Given the high structural similarity among catalytic domains within the USP family, achieving high selectivity represents a core challenge in inhibitor development. Current design strategies primarily leverage relatively variable regions surrounding the active site or unique allosteric pockets, alongside differences in spatial distribution among family members within specific organelles or protein complexes, this approach confers selectivity for particular USPs, enabling precise regulation of specific pathways while minimising off-target toxicity (Kazi et al., 2025).

### USP7 inhibitors

Several USP7 inhibitors have been developed to date, including representative compounds such as P5091 (as shown in Fig. 3B), HBX19818 (as shown in Fig. 3C), and GNE-6776 (as shown in Fig. 3D), P5091 demonstrated significant immunomodulatory effects in lung cancer models, reducing IL-10 levels while elevating IFN-γ and TNF-α levels, and enhancing the cytotoxicity of both CD4+ and CD8+ T cells (Dai et al., 2020). HBX19818 inhibits the deubiquitination of polyubiquitinated p53 by USP7, providing experimental evidence for its role in stabilising the p53 protein and exerting tumour-suppressing effects (Reverdy et al., 2012). Furthermore, our team demonstrated that USP7 inhibitors HBX41108 and GNE-6776, either alone or in combination with AMG510 (as shown in Fig. 3E), more effectively suppressed the proliferation of AMGR cells, this approach may represent an effective therapeutic strategy for inhibiting NSCLC cell proliferation and overcoming AMG510 resistance, particularly in KRAS G12C-mutated NSCLC (Huang et al., 2024). Concurrently, the new generation of USP7 inhibitors demonstrates enhanced potency and selectivity. The inhibitors FT671 (as shown in Fig. 3F) and FT827 (as shown in Fig. 3G) exhibit high-affinity and high-specificity inhibition of USP7 *in vitro* and in human cells. Co-crystallisation structures reveal that both compounds target a dynamic pocket near the catalytic centre of the USP7 autoinhibitory apolipoprotein structure. *In vivo*, FT671 destabilises USP7 substrates including MDM2, elevates p53 levels, drives transcription of p53 target genes, induces expression of the tumour suppressor gene p21, and ultimately inhibits tumour growth in mice (Schauer et al., 2020; Turnbull et al., 2017).

### USP14 inhibitors

IU1 (as shown in Fig. 3H), as the first reported selective inhibitor of USP14, binds to USP14, inducing a conformational change in the BL2 loop, this inhibits its deubiquitinating activity, enhances proteasomal degradation, suppresses lung cancer cell proliferation, and induces apoptosis (Srinivasan et al., 2023). Furthermore, USP14 acts as a regulator of the primary double-strand break repair pathway in non-small cell lung cancer (NSCLC). Ionizing radiation treatment increases USP14 levels and double-strand break recruitment in NSCLC cell lines, pharmacological inhibition of USP14 using IU1 enhances radiosensitivity in NSCLC cell lines (Sharma & Almasan, 2020). b-AP15 (as shown in Fig. 3I) recognises the 19S proteasome, inhibiting the activity of the deubiquitinating enzymes USP14 and UCHL5 associated with the 19S regulatory particle, this leads to the accumulation of polyubiquitin chains, thereby suppressing the proteasome’s degradation function, ultimately, it induces cell death by activating the cathepsin-D-dependent lysosomal apoptosis pathway (Chitta et al., 2015).

### Other USP member inhibitors

WP1130 (as shown in Fig. 3J) is a commonly used USP9X inhibitor that efficiently degrades ALDH1A3 and exerts a marked inhibitory effect on the growth of orthotopic xenografts derived from glioblastoma stem cells (Chen et al., 2019). Moreover, Gambogic acid (NGA) (as shown in Fig. 3K), an active component of garlic, also acts as a USP9X inhibitor, It significantly suppresses the proliferation of osteosarcoma cells through ubiquitin-proteasome-mediated degradation of SOX2 in both *in vitro* and *in vivo* experiments, whilst exhibiting minimal toxic side effects (Chen et al., 2020). ML323 (as shown in Fig. 3L) is a novel USP1/UAF1 deubiquitinating enzyme complex inhibitor exhibiting reversible nanomolar-level inhibitory activity and outstanding selectivity towards USP1/UAF1, furthermore, ML323 potentiates the cytotoxicity of cisplatin and increases endogenous monoubiquitination levels of PCNA and FANCD2 (Dexheimer et al., 2010). The secoiridoid glycoside gentiopicroside (as shown in Fig. 3M), acting as a specific inhibitor of USP22, attenuates the immunosuppressive function of Tregs by downregulating Foxp3 expression, concurrently, it suppresses tumour PD-L1 expression, thereby enhancing anti-tumour immunity and inhibiting tumour growth in mice with lung adenocarcinoma (Lu et al., 2024b).

**Figure 4:**
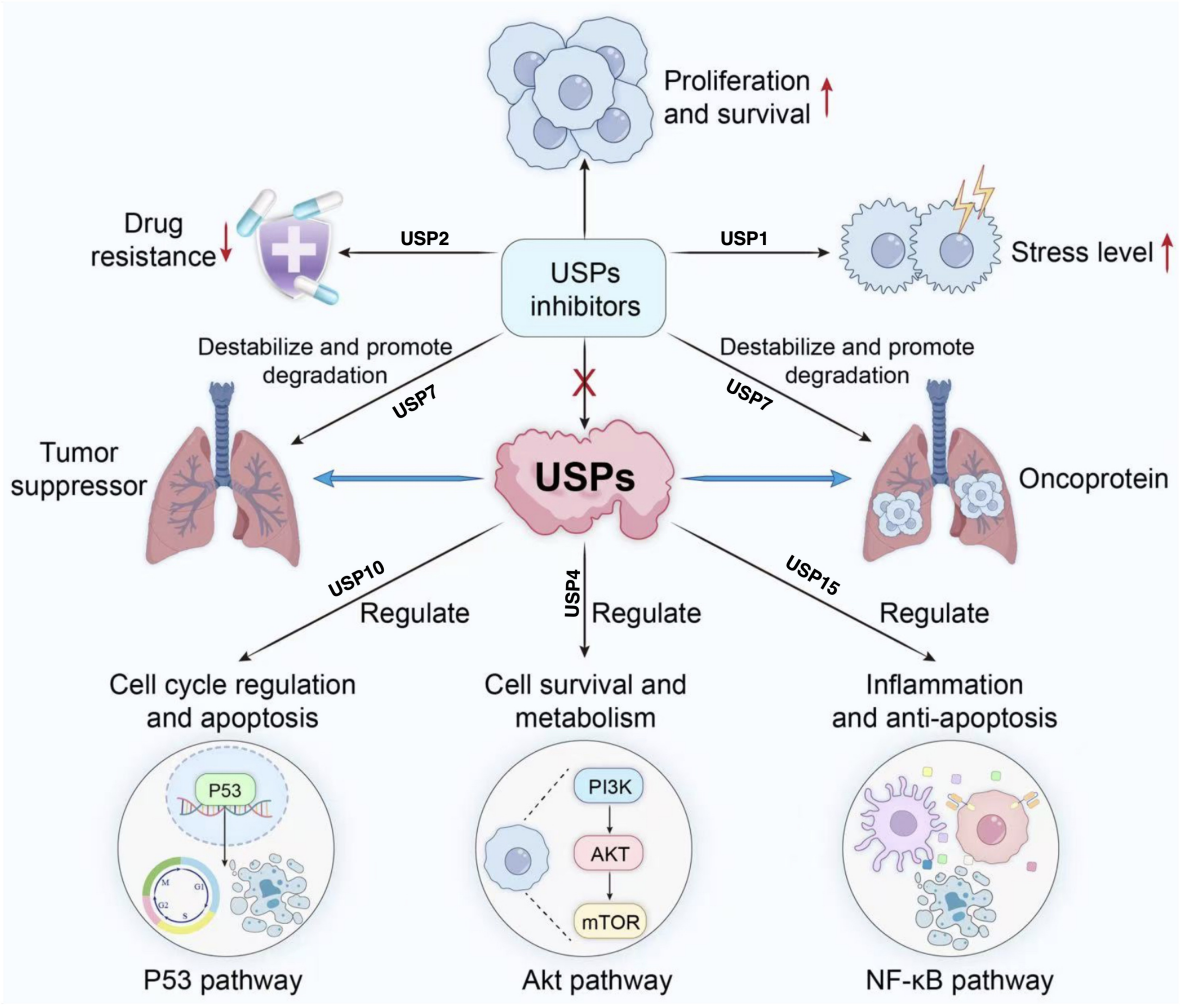
Role of USP inhibitors in cellular signaling pathways and drug resistance. USPs regulate tumour suppressor proteins and oncoproteins, participating in processes including cell cycle progression, survival and metabolism, inflammation, and anti-apoptosis. They act upon pathways such as p53, Akt, and NF-κ (B) USP inhibitors promote the degradation of associated proteins, thereby inhibiting proliferation and survival, reducing drug resistance, and alleviating stress responses.

Based on existing research, developing selective inhibitors targeting specific deubiquitinating enzymes represents another precision strategy. These enzymes exhibit preferences for particular ubiquitin chain types; for instance, members such as USP35 and USP38 tend to remove (Lysine residue at position 48 of the ubiquitin molecule)—linked ubiquitin chains from substrate proteins, thereby stabilising oncogenic proteins like BIRC3 and KLF5 and inducing drug resistance, conversely, USP10 and USP38 also participate in editing K63-linked ubiquitin chains, regulating the PTEN/AKT/mTOR pathway and DNA repair processes respectively (Gao et al., 2023; Hu et al., 2017). Furthermore, USPs participate in regulating key signalling pathways such as p53 and NF-κB, as illustrated in Table 2. Inhibiting USPs may disrupt these pathways, potentially impeding the proliferation and survival of lung cancer cells (Jiang et al., 2025; Li et al., 2024b; Pan & Deng, 2025; Wei et al., 2025). USP inhibitors may also induce apoptosis by elevating cellular stress levels and potentially reverse chemotherapy resistance in lung cancer cells. As illustrated in Fig. 4, this offers novel therapeutic avenues for intervening in tumour proliferation, metastasis, and treatment resistance (Chen, Liu & Zhou, 2021).

**Table 2:** The role of other USPs in lung cancer. This table summarises the ‘promoting’ effects of various deubiquitinating enzymes (such as USP1, Ubiquitin-Specific Peptidase 2 (USP2), USP18, Ubiquitin-Specific Peptidase 32 (USP32)) in lung cancer. By stabilising specific target proteins, they respectively regulate NK cell cytotoxicity, the p53 pathway, ferroptosis, and key signalling pathways such as RAF-MEK-ERK, thereby influencing tumour cell proliferation, migration, apoptosis, and invasion processes.

Deubiquitinating enzyme	Promote/ inhibit	Regulatory mechanisms in lung cancer	Relevant channels	References
USP1	Promote	In small cell lung cancer, USP1 regulates NK cell-mediated cytotoxicity, activation, and immune response pathways through deubiquitination	USP1/NK	Jiang et al. (2025)
USP2	Promote	In lung adenocarcinoma, it influences the cell cycle and apoptosis by regulating the p53 pathway, induces epithelial-mesenchymal transition (EMT), activates the MAPK/ERK signalling pathway, and promotes MMP2/VEGF-mediated invasion and metastasis	P53/p21/p27 EMT/MMP2/ VEGF MAPK/ERK SRC	Wei et al. (2025)
USP18	Promote	By deubiquitinating and stabilising the POU4F1 protein, transcription of PRKAA2 is promoted, forming the USP18/POU4F1/PRKAA2 axis that regulates proliferation, migration, apoptosis, and ferroptosis in lung adenocarcinoma cells	USP18/POU4F1/PRKAA2 Ferroptosis pathway	Pan & Deng (2025)
USP32	Promote	By deubiquitinating and stabilising the BAG3 protein, the RAF-MEK-ERK signalling pathway is activated, thereby accelerating the proliferation, migration and epithelial-mesenchymal transition (EMT) processes in non-small cell lung cancer cells	USP32/BAG3/RAF/ MEK/ERK	Li et al. (2024a), Li et al. (2024b) and Li et al. (2024c)

## Conclusions

Lung cancer, as the leading cause of cancer-related deaths globally, presents complex molecular mechanisms and high treatment resistance, making the identification of novel therapeutic targets an urgent priority. USPs regulate the stability of multiple key proteins through deubiquitination, thereby influencing proliferation, metastasis, apoptosis, and drug resistance in lung cancer cells. Research indicates that USPs such as USP7, USP9X, USP10, USP14, USP22, USP35, USP38, and USP39 exhibit multifaceted functions in lung cancer, engaging with multiple signalling pathways and biological processes.

USP7 promotes proliferation and drug resistance in lung cancer cells by stabilising key proteins such as ERβ, c-Abl, SMAD3, and KRAS. USP9X, meanwhile, influences apoptosis and radiotherapy resistance in lung cancer cells by regulating the deubiquitination of KDM4C and REV1. USP10, USP14, and USP22 respectively play crucial roles in cellular metabolism, signal transduction, and the immune microenvironment, thereby influencing lung cancer progression and therapeutic response. USP35, USP38, and USP39 exert effects on lung cancer through cell cycle regulation, inflammatory responses, and mitochondrial function.

Existing research indicates that USP inhibitors can effectively suppress the growth and metastasis of lung cancer cells, potentially reversing their resistance to chemotherapeutic agents. Despite significant advances in USP research within lung cancer, important limitations remain. Firstly, most studies have centred on USP7 and USP22, lacking systematic analysis of the USP family’s overall regulatory network and its synergistic or antagonistic interactions. Secondly, existing inhibitors generally suffer from insufficient selectivity and clinical toxicity issues; for instance, USP7 inhibitors may simultaneously affect the bidirectional regulation of p53 and MDM2 (Wang et al., 2019). Furthermore, the functional heterogeneity of USPs across different driver gene mutation subtypes—such as EGFR-mutant *versus* KRAS-mutant types—remains unexplored, potentially compromising the universality of targeted therapeutic strategies (Basar, Beck & Werner, 2021).

In summary, the molecular mechanisms of USPs in lung cancer and their potential as therapeutic targets offer new avenues for precision treatment of the disease. Future research should continue to delve into the functions and regulatory mechanisms of USPs, with the aim of achieving breakthroughs in clinical applications.

## Additional Information and Declarations

### Data Availability

The following information was supplied regarding data availability: This is a literature review.
